# Epidemics and Their Everyday Causes

**Published:** 1858-04

**Authors:** W. I. Cox


### EPIDEMICS AND THEIR EVERY DAY CAUSES.

By W. I. COX, M.R.C.S.

I PROPOSE, in the present paper, to call the attention of the readers of the SANITARY REVIEW to some of the many deleterious agents which prevail in numerous localities, as bearing on the development and diffusion of epidemic disease.

The influence of the various seasons of the year—of atmospheric and terrestrial electricity upon the human organism—the effect of the electric tension on the production or prevention of disease—the geographical conditions of epidemics, and the meteorological states which are generally found associated with the outbreak of pestilences—all these are unquestionably subjects, the importance of which it would be difficult to overestimate, and which well merit the most diligent and patient investigation. But, up to the present time, they remain, as ever, shrouded in doubt and incertitude. They are too much *in nubibus*, too remote, to be available for practical application. It is not so with the matters I am now about to discuss. For, if chemical and physiological science has proved anything, if it has succeeded in placing any fact in an unassailable position, it is the following;—that animal and vegetable emanations are a fruitful source of zymotic disease; that these noxious agents operate as causes in regard to the whole class of epidemics; that what will in one place develope the exciting cause of typhus, will in another produce scarlatina, small-pox, or cholera. In fact, medical men, who practise much among the poor, can generally, when any epidemic is impending, predict with tolerable certainty the localities where it will appear; where its type will assume its greatest malignity; where its victims will be most numerous, and its mortality most deplorable. If this be true, and we find on inquiry that these noxious agents exist and flourish unchecked and unheeded in too many places amongst us; that in such spots the *materies morbi* is never absent, but that there is kept up a perpetual hot-bed, so to speak, ready at all times for the reception and growth of the fatal germs of zymotic poison; we must surely admit that these aforesaid predisposing and developing causes of disease are far more important to us than the proximate cause; and that it would be much more advantageous and expedient to direct our attention to remediable and removable influences, which can be shewn to exercise so vast a power over the spread of pestilence, than to bewilder ourselves with abstruse speculations concerning the absolute essence of zymotic poison, or the laws of its aërial passage from clime to clime: things which, even if comprehended, would doubtless remain evermore as far removed from the sphere of our control as the law of storms, or the velocity of the tidal wave.

If the non-professional reader ask, Do these hurtful agents still exist, and in number and force to justify a crusade? statistics from a hundred sources, parliamentary and medical, cry out a loud affirmative. They show that, in numerous localities, the poor are crowded in stifling dens, wherein the rich would not consent to keep their domestic animals; that in hundreds of places they are compelled to drink poisonous water, instead of the pure element which God gave for the use of men and beasts; that dirt, misery, ignorance, and depravity, reign supreme in scores of densely populated cities; and that the baneful gases, which constitute the *pabulum* of zymotic poison, are constantly being generated and concentrated in the homes of thousands in our favoured isle.

Statistics further show us, that epidemics ravage only those districts wherein there are *endemic* influences at work. Now these epidemic agents it lies within the scope of human power to lessen and remove.

I divide these local agents or causes of disease into two broad classes; 1. Those of a physical, and 2, those of a social or moral origin.

Under the first head, I shall consider,

1. Overcrowding.
2. Defective drainage and ventilation.
3. The use of foul water.
4. Privation of solar light.

Under the second division, I shall have to speak of,

1. Intemperance.
2. Filth.
3. Neglect of infantile life.
4. Unhealthy occupations.

### I. OVERCROWDING.

The poisoned atmosphere produced by the aggregation in close rooms of an excessive number of human beings, is one of the most influential agents in developing disease. Its pernicious qualities are not due alone or chiefly (as is very commonly supposed) to the increased amount of carbonic acid gas. They are attributable to a still more formidable agent, viz., the presence of effete organic matter, thrown off from the lungs and skin. This effete body is an albuminous compound in a state of rapid decomposition,* whence results the liberation of hydrosulphuric acid, and other noxious gases. Do not let us be deluded with the idea, that the miseries and evil results of overcrowding are confined to great cities, or towns of third and fourth-rate size. I have frequently encountered them in obscure and petty country villages, where are to be found ill-built, close, stifling cottages, and low lodging-houses, crammed to excess each night with human beings. The consequences are not less fatal to the health and morality of the inhabitants of such dwellings, than to those of densely populated towns. Evil is assuredly evil, find it wherever we may. As regards the moral enormities which result from such an abominable system (and which re-act, in their turn, on the physical constitution, by means of the recklessness and degeneracy they occasion) who can wonder at these? Where human creatures are shut up at night in the same fold, like the beasts of the field; with no distinction of sex—males and females, with no other tie between them than the link of a common nature, a common appetite, occupying the same stifling chamber, the same filthy and polluted couch—we cannot wonder at the revolting scenes which are sometimes brought to light.

* Experiments, which cannot of course be detailed in this place, have abundantly proved to me the truth of this assertion. They entirely confirm the results of those instituted by Dr. Angus Smith.

I will now proceed to give a few examples of the connection of overcrowding with the development of typhus, scarlatina, and cholera. I am aware that, in statements of this nature, it is necessary most carefully to guard against the philosophical empiricism of hasty conclusions from insufficient evidence. Many writers have traced, as they thought, a malady to its source, but have merely exhibited striking examples of causation, hastily and erroneously inferred from casual conjunction. This error will now be avoided as far as possible.

*Typhus.* In December 1848, and January 1849, I had the care of the sick poor in the northern district of the Poplar Union. No fever cases (excepting in two or three scattered instances) had occured in the district, I was informed, for two years previously—either in the town of Bow, or the adjacent village of Bromley. Two recently built streets (or rather parallel rows of houses) in the latter place became occupied with Irish families during the previous summer. As respects drainage, water-supply, and general arrangements, these streets were quite on a par with the rest of the village. About the middle of December typhus, of a very low type, appeared in three houses almost simultaneously, in the lowest of these streets. (They ran parallel to each other, being situated on a slight declivity). In an incredibly short space of time it had swept through both of them. The medical practitioner who had charge of the district was seized with the malady in its worst form, and died comatose in ten days. I then entered upon the duties. On the 8th of January, not a habitation in the lower street but had at least two of its inmates "down" in the fever; and in the upper street I had also twenty-eight fever cases under my care. In many of the houses two of the in-dwellers were lying comatose; and in four of these habitations, three persons in each were attacked at the same time. In all, fifty-three cases of fever occurred within the limits of these two streets between the middle of December and the beginning of March. The disease did not extend to the rest of the village, neither did it break out elsewhere within the district. Only one case occurred at Bow. This was in the person of a young woman, who, returning home from distant service in the commencement of February, stayed a few days with her parents, who lived in one of the streets above described; and afterwards went to reside at Bow, in the service of a respectable family, whose dwelling was dry and airy. She was seized with the fever the day after her removal. Now what was the state of things in these houses? The overcrowding was awful. Each house consisted of four rooms, about twelve feet square. An entire family lived and slept in each chamber. In one, I counted seven human beings, who occupied the same filthy couch,—a father, mother, three adult daughters, and two younger children! In a second room, six persons slept, viz., a widow, her two grown-up daughters, an adult son, and two young children! There were two beds, it is true, but no partition or curtain between the sexes. A third room contained five—a fourth the same. Not to tediously repeat the same fact, one room in any of these houses was a fair specimen of the whole. It is unnecessary to say, that they were indescribably foul, fetid, close, and disgusting.

In the above instance, we can have no hesitation in ascribing the concentration and severity of the fever-poison (if not, indeed, its actual development) to the vitiated atmosphere produced by the overcrowding. I made every inquiry, but was quite unable to trace the origin of the disease to any other source.

Whilst in Lancashire in 1855, the incumbent of the district adjoining that in which I resided, called my attention to the following fact. A lone cottage in his parish had been visited by fever of a low type every winter for some years past, carrying off, on each occasion, at least one of the inmates. No cause, he said, could be assigned for the visitation; nor did the disease prevail in the neighbourhood at such times. The cottage was provided with tolerably good drainage, and the character of its indwellers was that of average sobriety. "In short," this gentleman concluded, "I fancy you sanitary men would find yourselves at fault here for once." I visited the place with some curiosity. The house was a thatched cottage, very low-roofed, and with exceedingly small rooms; especially the bedrooms, which were not quite nine feet square by six feet in height. There was no cause for censure on the part of cleanliness. A middle-aged couple and one adult* son were the only inmates at the time of my visit (during the summer). But on inquiry, I discovered, that during the winter months they took in, as lodgers, a number of young men, varying from five to eight; and that these were all packed at night, with the son of the host, into the two small sleeping apartments just mentioned. So that four and sometimes five grown-up men were in the habit of sleeping, for months in succession, every night in a room containing about 480 cubic feet of air; having no fireplace, and no "practicable" window. These men left in the spring to procure agricultural employment at a

* Another younger son had died of the fever the previous winter.

distance. Here was, to me at least, a sufficient solution of the apparent mystery.

In another village in Lancashire I was called in to attend a young woman, who had been seized with low fever ten days previously. She was in a hopeless condition, and sank the next day. There were not, nor had there been, any other cases of fever in the village. On entering the room where she lay—a perfect den—I was struck with horror at the fœtor and the hideous picture which presented itself. This room (in a lodging-house) was about eleven feet square. It contained three beds; one contained my patient, and, up to the day previous to my visit, two other adult females; the second, two adult unmarried males; and the third was occupied by a man, wife, and child. The excretions of my patient had not been removed for twenty-four hours. There was no attempt at the preservation of decency. Another of the women fell ill of the fever; she was removed by her friends, and recovered.

*Cholera.* During the visitation of Asiatic cholera in 1849, I was resident for a considerable period of time in the district of Poplar, and was entrusted by Mr. Webb, the able and zealous union medical officer, with the chief charge of the poor in the Isle of Dogs. The western side of this delectable island, (or rather peninsula), called Millwall—by far the most populous portion—escaped the ravages of the epidemic tolerably well. Not more than seven fatal cases occurred; in fact, the town of Poplar, Blackwall, and contiguous district, suffered much more severely. The houses in this part of the island are decently built, drained, commodious, and by no means overcrowded with inmates. But on the eastern portion of the island (which lies over against Blackwall) and towards its lower part—not very far from the ferry to Greenwich—are, or were at the time of which I speak, several desolate-looking and miserable cottages; low-roofed, one-storied, having the aspect of pigsties rather than of human habitations. These wretched houses stood in a brickfield, which afforded employment to the persons living in them. They were crammed to excess with human beings. Four, five, and in some cases six adult men lived and slept in each room. These apartments were about ten feet square, by six feet in height. The walls were bare and damp; the floors mostly of mud. There was no thought of, or care for, ventilation. None of the casements would open. there was only one fireplace in each house. The pestilence soon made horrid havoc in these dreadful abodes. On my second visit, I saw in one house, three corpses; in a second two; in a third one; all victims of cholera since my first visit on the previous day. There also lay ill of the pestilence at the same time in these three cottages five more persons, three of whom subsequently died. The fetid effluvium proceeding from some of the sleeping chambers in these houses was sickening, and strikingly characteristic of human emanations from overcrowding and absence of pure air. In one close (I may almost call it air-tight) room, six full-grown men slept nightly. Now, supposing the period of occupation to be only six hours, and the room to be filled with pure air at the commencement of the night, (both which positions certainly could not be asserted *as facts* in this case), there should have been a capacity for containing 2520 cubic feet of air: viz., 70 feet an hour for each person, which is somewhat, indeed, below the proper amount. I say, *there should have been* this capacity; for there was no chimney—the window was hermetically sealed—and the very close-fitting door was kept fast shut by some females who slept in the adjoining room. Now what was actually the case? The chamber was 13 by 10 feet, and 6 feet in height, containing, of course, but 780 solid feet of air. In a somewhat similar den in another of these cottages, in which slept a man, his wife, and child, and two other men—a tattered curtain being drawn across the room, separating the beds—I found, that instead of the due supply of pure air for the maintenance of healthy respiration, these unhappy persons (the child not even being taken into the account) were limited to about 25 feet of air an hour each.

The mind revolts at such horrible statements; yet such is common in the habitations of the poor in town and country. In consequence of the scantiness of house-accommodation and high rents, the labouring class are in too many places compelled to eke out their narrow means by underletting their wretched dwellings, or cramming them with as many lodgers as they can obtain.

*Scarlatina.* In the summer of 1846, this malady was epidemic in various metropolitan districts; but mild in type, and amenable to simple treatment. On a sudden, however, it broke out in a dismal court at the back of Covent Garden, (close to Hart Street, but I now forget the exact locality, which is unfortunately not stated in my notes), with fearful and uncontrollable malignity. I was requested to visit the scene, and take the medical charge of the sufferers. The state of things in the three houses to which the violence of the epidemic was chiefly confined in this court, was appalling. The description of one horrible room in the central house must suffice, as presenting an average picture of the whole. This apartment (on the third floor) was about 12 feet square, filthy in the extreme. In one corner lay a woman, who had been confined about a fortnight, on a wretched pallet with her luckless infant. In another corner lay two girls, of eight and ten years of age, huddled together on a kind of rug. They were the daughters of the lying-in woman, and were evidently near death. At the other end of the room were four more children, varying in age from two to nine years, in different stages of the disease. *Their* mother was absent at the time of my visit. They were all cases of genuine *scarlatina maligna*—intense throat affection and prostration at the very onset of the malady. The two girls first mentioned died the next day, and two of the other children three days afterwards. There were altogether nineteen cases in the three houses, whereof ten terminated fatally. Now I can *confidently* attribute this fearful mortality to the overcrowding ; as, although the disease prevailed extensively in the neighbouring streets, it did not assume the same malignancy of type, and yielded to remedial measures. The overcrowding and dirt of this court had long been notorious. I may add, that both myself and a friend who accompanied me on my visits, and shared the professional duties with me, contracted the disease, and narrowly escaped with our lives.

II. DEFECTIVE HOUSE DRAINAGE AND VENTILATION.

It is unnecessary to dilate on the fact, now generally admitted, of the immediate connection of disease of various kinds with neglected drainage. Yet the bulk of the community are inclined to the very erroneous supposition, that this evil is confined almost entirely to large towns and squalid districts. But such an impression is far from the truth. Side by side with our wealth of power and enterprise we find, in this respect, elements of fatal weakness—of perdition—which can scarcely perhaps be paralleled among savage nations. Tall rows of handsome houses, in flourishing cities, too often conceal deformities worse than hideous—a repulsive background of ghastly squalor to the brilliant picture. Here, within a stone's throw of the dwellings of rank and fashion, are the harvest-fields of death. Stately dwellings, in country towns, are too frequently ill-drained, and worse ventilated. Whenever these evils exist, there also will appear, from time to time, their deplorable and certain consequences. The docks at Liverpool, filled with stagnant water, and receiving the refuse of hundreds of ships, proved to the adjacent neighbourhood during the epidemic of 1849, as fatal a neighbour as the tanks of filthy water under the wretched huts of Mevagissey.

The non-medical reader may not perhaps be aware, that the deleterious emanation from cesspools and ill-drained privies, consists chiefly of sulphuretted hydrogen. This gas is of so deadly a nature, that an atmosphere containing only 1-800th part will speedily destroy a sparrow. I found in the course of my experiment on this body, that a mouse was soon poisoned when confined in a large vessel of air impregnated with 1-600th of its volume. Professor Wagner states, that he found 1-400th ultimately fatal to a horse. And yet I have been in human habitations wherein the lead of the window-sashes, and all the exposed painted portions, were black from the action of this gas, which was constantly being given off in large quantities from a cesspool close by. Fever had been a frequent visitor in these houses.

The town of Birmingham has hitherto enjoyed very great immunity from *cholera.* Scarcely a case occurred within its precincts in the first visitation of 1832; and but very few in 1849 and 1853. This cannot be attributed to anything artificial in the condition of the inhabitants; to their superior sobriety, cleanliness, or physical power of resisting disease. What then is most probably the cause? I conclude that we may fairly consider it to be due to the favourable circumstances of the town as regards natural drainage. It stands on a light and porous sand-stone, and also abounds in steep inclinations. The perambulator on foot finds plenty of "up and down work" within its compass. As respects other sanitary matters, the town was then on a par with other large places, which suffered severely from the ravages of the epidemic.

The town of Hindley, in Lancashire, suffered fearfully from the visitation of the cholera in 1849. Out of a population of about 4000, it swept off, I was informed, 180 in the course of a fortnight or three weeks. No medical man, however, who visits this filthy town, will wonder at this, nor at the fact that almost *every epidemic* therein very often assumes a malignant type. I shall have to refer to this further on. A large portion of the houses are situate around cotton-mills, wherein the occupiers labour. More than fifty of these houses have literally *no drainage,* and *no water-supply.* There are no necessaries attached to them. The men use the public privies provided by the mills; the women and children extemporise, *lieux d'aisance,* in chamber-utensils, which are being emptied, at all times and seasons, into a polluted stream, which flows sluggishly in front of the foremost row of houses. It is sad to conceive the depravity of sentiment which gradually grows up to tolerate the presence—the constant sight of the contact—

of human *egestæ.* In fact, there is no effort made, in some instances, to avoid or remove the most loathsome of excrementitious matters. Apart from the horrible *physical* contamination—a hot-bed for disease—the *moral* contamination is conspicuous. Let any one who doubts this, visit either the place I have named, (which is unhappily the type of a large number of small towns in the north), or the back streets of Glasgow, Liverpool, or London. Where such a dreadful state of things exists, the physical evil never stands alone. With it, we are sure to find associated every kind and degree of improvidence and licentiousness. There is no idea among such people of the laws of health ; hence consumption of unwholesome food, and indulgence in intoxicating liquors, prevail. Cleanliness of person and habitation is altogether disregarded. In proportion to the degradation, so, alas! is the tendency to adhere to it ; to sink deeper and deeper into the mire of pollution ; to aggregate and multiply in numerical strength, and in the materials for mischief and disorder, the seeds of disease and crime. The public necessaries above alluded to have seldom any connection with drain or sewer. They simply consist of a pit or cesspool, which, when overflowing, and not before, is partially emptied. There is always an offensive stench in the vicinity of these abominations, and *fever is scarcely ever absent from it.* Partly owing to the natural influence of the daily association with places so filthy, and partly to the entire absence of anything like supervision, these public privies become the medium of the most disgusting profligacy.

*Deficient Ventilation* generally accompanies the evils, and aggravates the consequences, resulting from neglect of drainage. But it often exists *per se,* among classes generally supposed to enjoy exemption from such ills, and to an extent that could not be known save by actual experience. Atmospheric impurity is not confined to the abodes of the wretched. In the drawing-rooms of the great ; in temples of pleasure or worship, proper ventilation is not the rule, but the exception. I have entered dissenting chapels in the midst of the service, both in town and country, and been immediately sickened and assailed with the sense of foul impurity from the concentrated reek of organic effete vapours. In tailors' and milliners' workrooms this evil is of a deplorable magnitude. I once visited a room (underground) in a house in London, wherein thirty-four young women worked for twelve, sometimes fifteen hours a day. It was about sixteen feet square ; it had no fireplace, or artificial means of ventilation. The door and windows were kept fast shut. The sense of foul air was so offensive, that I

would rather have dined and slept in a good dissecting-room. Most of the poor young girls were pallid, anæmic, and scrofulous. One, I was informed, was then at home lying ill of fever. The greatest ignorance prevails on this matter, even among the educated classes. I lately attended a lady who suffered from severe headache and dulness of the mental faculties. I prescribed for some time in vain, until, at last, I discovered the cause of her malady. She slept in a very small chamber, as close as a box, and blocked up with furniture. The chimney had been carefully stuffed up with straw to keep out the cold! I cleared this out, and my patient had no more headaches. The affection had always come on during the night, and wore off gradually in the day-time.

---

